# Complete Genome Sequence of a Novel Variant Strain of Peste des Petits Ruminants Virus, China/XJYL/2013

**DOI:** 10.1128/genomeA.00762-14

**Published:** 2014-10-09

**Authors:** Jingyue Bao, Qinghua Wang, Yongqiang Zhang, Chunju Liu, Lin Li, Zhiliang Wang

**Affiliations:** China Animal Health and Epidemiology Center, Qingdao, Shandong, China

## Abstract

Here, we announce the complete genome sequence of a novel variant strain of peste des petits ruminants virus, termed China/XJYL/2013. The genome is 15,954 nucleotides long with a 6-nucleotide insertion in the 5′ untranslated region of the F gene. This strain is phylogenetically classified as a lineage IV virus.

## GENOME ANNOUNCEMENT

Peste des petits ruminants virus (PPRV) is a highly contagious viral pathogen of small ruminants (1) that is endemic across much of Africa, the Middle East, and Asia, and it has recently emerged in China (2, 3). PPRV is regarded as a significant hurdle to the development of sustainable agriculture, and it causes economically significant mortalities (3). PPRV belongs to the genus *Morbillivirus* in the family *Paramyxoviridae*. As with all morbilliviruses, only one serotype of PPRV exists serologically, although genetically, its isolates can be grouped into four lineages. The genome of PPRV is a nonsegmented, negative-sense, and single-stranded RNA. Currently, full-genome sequence data are available for 9 PPRV strains from different parts of the world, with a genome size of 15,948 nucleotides (nt) (4–7). Here, the complete sequence of a novel variant PPRV strain, China/XJYL/2013, was sequenced and analyzed.

In November 2013, an outbreak of PPR was detected in a goat farm located in the Yili region of Xinjiang province in China. Tissue samples from lymph nodes were collected from a sick 1-year-old goat showing signs of disease. Viral RNA was extracted and used directly for viral genome determination. Fourteen pairs of oligonucleotide primers were designed to amplify 14 overlapping fragments of the China/XJYL/2014 strain by reverse transcription-PCR. The PCR products were puriﬁed and sequenced with an ABI 3730XL genome sequencer (Applied Biosystems, USA). The genome termini were determined using 3′/5′ rapid amplification of cDNA ends (RACE) (7). A total of 156 sequences were assembled to yield the complete genome sequence (DNAStar, Inc.), with an average of 6.68-fold coverage at each nucleotide position.

The genome size of China/XJYL/2013 is different from that of previously published PPRV isolates, at 15,954 nucleotides, with a 6-nt insert at nucleotide 5215 within the 5′ untranslated region (UTR) of the F gene. China/XJYL/2013 has an identical genome organization with other PPRV isolates, including the nucleocapsid (N), phosphoprotein (P/C/V), matrix (M), fusion (F), hemagglutinin (H), and the large polymerase (L) proteins. Genome and antigenome promoter regions, gene start and stop sequences, and intergenic trinucleotides were present, as expected. At the nucleotide level, China/XJYL/2013 shares 97.4% homology with Tibet/30/07 (GenBank accession no. FJ905304) and Tibet/07 (accession no. JF939201), 97.3% with Tibet/Bharal/2008 (5), 96.7% with Turkey/2000 (7), 95.4% with Sungri/96 (accession no. KF727981), 96.4% with Morocco/2008 (4), 91.7% to 91.8% with Nigeria/75/1 (accession no. X74443), and Nigeria/76/1 (6), and 88% homology with ICV/89 (6). A direct comparison of China/XJYL/2013 with Tib/30/07 demonstrated 17 mutations in P (3.34% variation in the amino acids of P protein), 11 mutations in H (1.81% variation), 7 mutations in F (1.28% variation), 6 mutations in N (1.14% variation), 23 mutations in L (1.05% variation), 10 mutations in C (5.65% variation), and 15 mutations in V (5.03% variation). Importantly, the mutations in the P and associated accessory proteins may be important for immune modulation. Phylogenetic analysis of the partial F or N gene sequence by MEGA 4.0 suggests that China/XJYL/2013 belongs to lineage IV, being closely related to viruses circulating in Pakistan and Tajikistan. Further studies are essential in order to discover whether the genome length of 15,954 nt is a common characteristic of the PPRV isolates circulating in the mid-Asia region.

### Nucleotide sequence accession number.

The full-genome sequence of PPRV isolate China/XJYL/2013 has been deposited in GenBank under the accession no. KM091959.

## References

[1] Gibbs EP Taylor WP Lawman MJ Bryant J 1979 Classification of peste des petits ruminants virus as the fourth member of the genus *Morbillivirus*. Intervirology 11:268–274. 10.1159/000149044 457363

[2] Banyard AC Parida S Batten C Oura C Kwiatek O Libeau G 2010 Global distribution of peste des petits ruminants virus and prospects for improved diagnosis and control. J. Gen. Virol. 91:2885–2897. 10.1099/vir.0.025841-0 20844089

[3] Wang Z Bao J Wu X Liu Y Li L Liu C Suo L Xie Z Zhao W Zhang W Yang N Li J Wang S Wang J 2009 Peste des petits ruminants virus in Tibet, China. Emerg. Infect. Dis. 15:299–301. 10.3201/eid1502.080817 19193278 PMC2657621

[4] Muniraju M El Harrak M Bao J Ramasamy Parthiban AB Banyard AC Batten C Parida S 2013 Complete genome sequence of a peste des petits ruminants virus recovered from an alpine goat during an outbreak in Morocco in 2008. Genome Announc. 1(3):e00096-13. 10.1128/genomeA.00096-13 23661470 PMC3650429

[5] Bao J Wang Q Parida S Liu C Zhang L Zhao W Wang Z 2012 Complete genome sequence of a peste des petits ruminants virus recovered from wild bharal in Tibet, China. J. Virol. 86:10885–10886. 10.1128/JVI.01503-12 22966182 PMC3457324

[6] Chard LS Bailey DS Dash P Banyard AC Barrett T 2008 Full genome sequences of two virulent strains of peste-des-petits ruminants virus, the Côte d’Ivoire 1989 and Nigeria 1976 strains. Virus Res. 136:192–197. 10.1016/j.virusres.2008.04.018 18541325

[7] Bailey D Banyard A Dash P Ozkul A Barrett T 2005 Full genome sequence of peste des petits ruminants virus, a member of the *Morbillivirus* genus. Virus Res. 110:119–124. 10.1016/j.virusres.2005.01.013 15845262

